# Azathioprine Hypersensitivity Syndrome Mimicking an Infection in a Systemic Lupus Erythematosus Patient: A Case Report

**DOI:** 10.7759/cureus.86085

**Published:** 2025-06-15

**Authors:** Tania Paola Lujan Chavarria, Carlos Esteban Giraldo, Natalia Andrea Uribe Ruiz, Cynthia Chavarría Muriel, Adriana Lucia Vanegas-García

**Affiliations:** 1 Internal Medicine, San Vicente Fundacion Hospital, Medellin, COL; 2 Internal Medicine, University of Antioquia, Medellin, COL; 3 Dentistry, Vision of the Americas University Institution, Medellin, COL; 4 Rheumatology, San Vicente Fundacion Hospital, Medellin, COL

**Keywords:** azathioprine, case report, fever, hypersensitivity syndrome, systemic lupus erythematosus

## Abstract

Azathioprine (AZA) is used in clinical practice as a steroid-sparing medication to treat diverse systemic autoimmune diseases, such as inflammatory bowel disease, rheumatoid arthritis, and systemic lupus erythematosus. The adverse effects of AZA use include azathioprine hypersensitivity syndrome (AHS). AHS is a rare entity, but it can lead to fatal outcomes if not identified early. Fever and abdominal pain, while resembling other diseases, can be a sign of this disease. This paper discusses the case of a patient diagnosed with systemic lupus erythematosus who experienced AHS.

## Introduction

Azathioprine (AZA) suppresses the immune system by inhibiting purine synthesis, disrupting DNA and RNA production, and impairing lymphocyte function [1]. This action helps to prevent organ rejection and reduce inflammation in autoimmune conditions. While other immunosuppressive agents are more efficacious, AZA is still used as one of the medications for the treatment of lupus nephritis due to its better tolerability and safety and is also prescribed for other systemic lupus erythematosus (SLE) indications [2]. Although AZA is generally considered safe, the adverse effects of this medication are not negligible. Azathioprine hypersensitivity syndrome (AHS) is an idiosyncratic drug reaction and non-dose dependent. Clinical features include constitutional symptoms with or without a cutaneous reaction and, less commonly, liver and renal dysfunction, hypotension, and shock [3]. It has a low incidence (about 2% of patients), but it is a potentially life-threatening entity involving multiple organs. In many cases, the symptoms may be similar to a disease relapse or may mimic sepsis symptoms, thus becoming a diagnostic challenge [4,5].

There are relatively few cases of AHS with fever and abdominal pain as symptoms reported in the literature [6,7]. Thus, in this article, we report a case of AHS in a patient who had fever and abdominal pain mimicking an infection.

## Case presentation

A 22-year-old woman diagnosed with SLE whose diagnosis was reached due to the presence of leukopenia, photosensitivity, arthritis, immunological compromise (antinuclear antibody (ANA) test with a 1:2560 titer and a speckled pattern and high anti-RNP and anti-Sm), and deep vein thrombosis. The patient was receiving chloroquine, apixaban, and nifedipine treatment. After being hospitalized 17 days prior to admission due to a diagnosis of pericarditis and pleural effusion in the context of lupus serositis, the tests registered the first positive lupus anticoagulant. Hence, she was discharged to continue treatment with prednisolone (30 mg daily), hydroxychloroquine (200 mg daily), warfarin (5 mg daily), calcium citrate+vitamin D (1500+200 IU daily), and AZA (50 mg every 12 hours).

**Figure 1:**
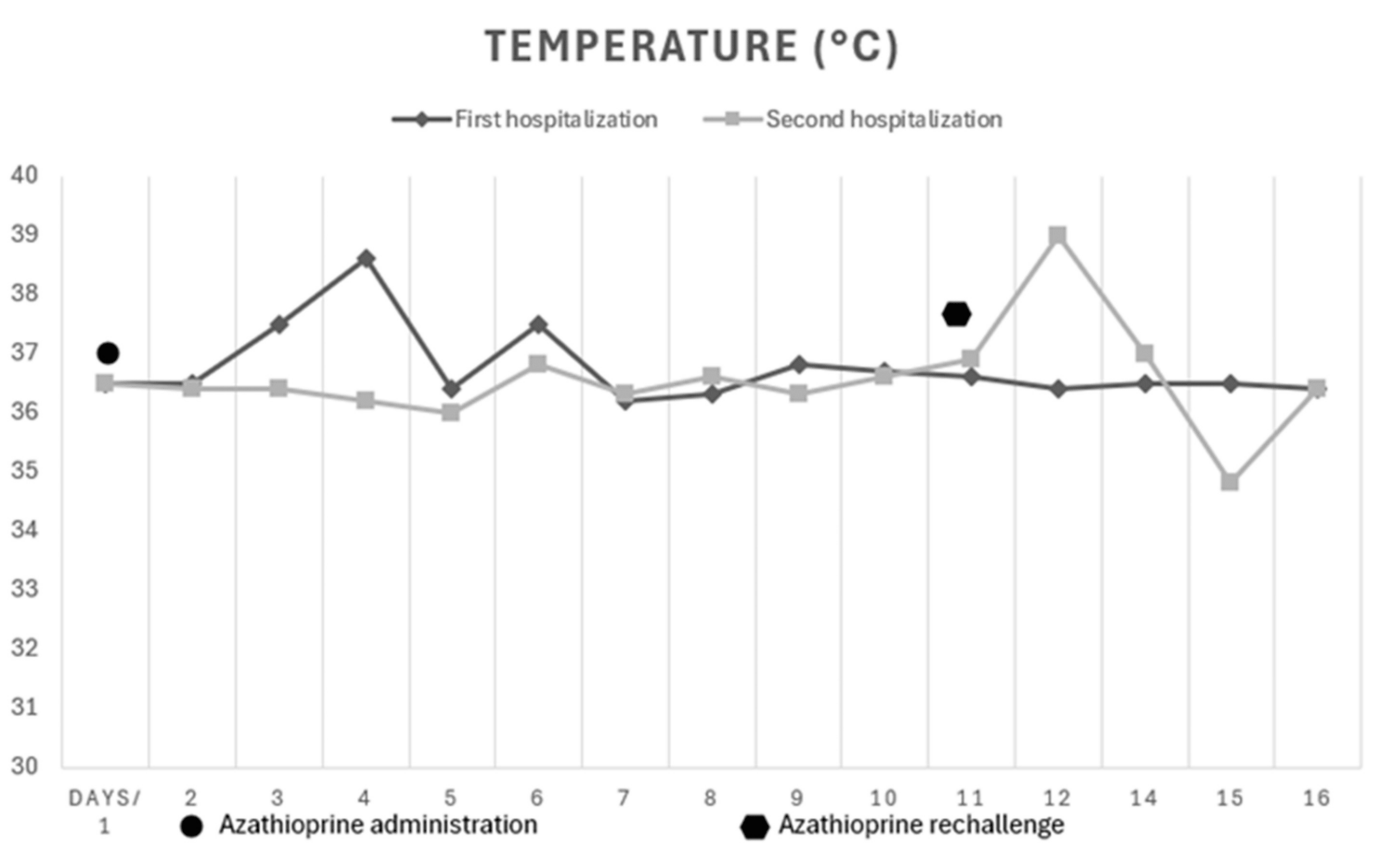
Detailed temperature timeline in the first and second hospitalization: azathioprine discontinuation, resolution, rechallenge, and recurrence

She was readmitted four days post-discharge (two days with AZA) in the emergency room displaying objective fever (temperature >38.7°C), chills, and general malaise, along with odynophagia, constant low-intensity hypogastric pain, and erythema on her hands and knees that appeared along with fever. Physical examination yielded the following results: blood pressure of 110/74 mmHg, heart rate of 142 beats per minute, body temperature of 38.6°C, and globally decreased vesicular breath sounds and tenderness to palpation in the hypogastrium (see Figure 1: first hospitalization).

Paraclinical tests showed that the renal function, liver profile, hemoglobin complements, and leukocytes were normal, platelets were at 129000 per mm^3^, and C-reactive protein was at 20 mg/L (see Table 1).

**Table 1:** Summary of laboratory test results: the patient had mild elevated inflammatory markers

	First hospitalization	Second hospitalization	Reference (units)
Hemoglobin	13.5	11.8	12-16 (g/dL)
Leukocytes	6800	3500	4500-11000 (/µL)
Neutrophils	73.5	63.1	45-65 (%)
Lymphocytes	17.6	29.1	30-40 (%)
Platelets	266000	248000	150000-450000 (/µL)
Urea nitrogen	11.4	7.7	9-23 (mg/dL)
Creatinine blood	0.64	0.57	0.57-1.11 (mg/dL)
C-reactive protein	4	3.7	0.4-1 (mg/dL)
Sedimentation rate	20	10	0-15 (mm)
Complement C3	102.7	119	82-160 (mg/dL)
Complement C4	26.7	28.7	12-36 (mg/dL)
Total bilirubin	0.61	0.51	0.2-1.2 (mg/dL)
Direct bilirubin	0.17	0.16	0-0.3 (mg/dL)
Alanine transaminase (ALT)	25	37	9-52 (U/L)
Aspartate aminotransferase (AST)	42	42	14-36 (U/L)
Alkaline phosphatase	85	42	38-116 (U/L)
Procalcitonin	-	0.71	0-0.1 (ng/mL)

**Figure 2:**
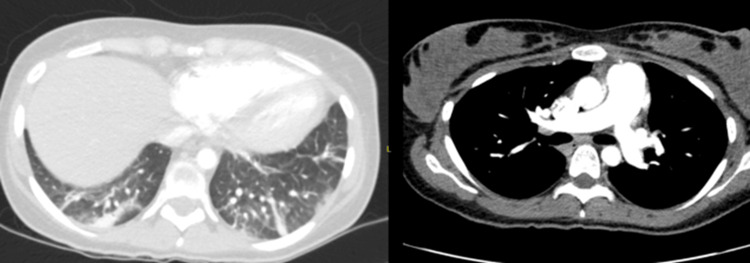
The chest angiotomography ruled out pulmonary embolism and only showed basal atelectasis. She did not have pneumonia

SARS-CoV-2 infection, bacteremia, and urinary tract infection were ruled out after negative blood and urine cultures. Prednisolone was administered at a stress dose, and other immunosuppressants were discontinued from the treatment. The chest angiotomography ruled out pulmonary embolism and only showed basal atelectasis (see Figure 2).

During the stay in the emergency room, she had tachypnea and decreased pulse oximetry, requiring supplementary oxygen therapy. Empirical antibiotic therapy started with piperacillin-tazobactam due to a suspected diagnosis of community-acquired pneumonia. A bronchoscopy with bronchoalveolar lavage was performed, and negative cultures of aerobes, fungi, and mycobacteria were reported. She completed seven days of antibiotic therapy with significant improvement of her symptoms and was discharged to continue therapy with AZA (50 mg every 12 hours), hydroxychloroquine (200 mg daily), prednisolone (20 mg daily for 15 days), and calcium citrate+vitamin D (1500+200 IU daily).

**Figure 3:**
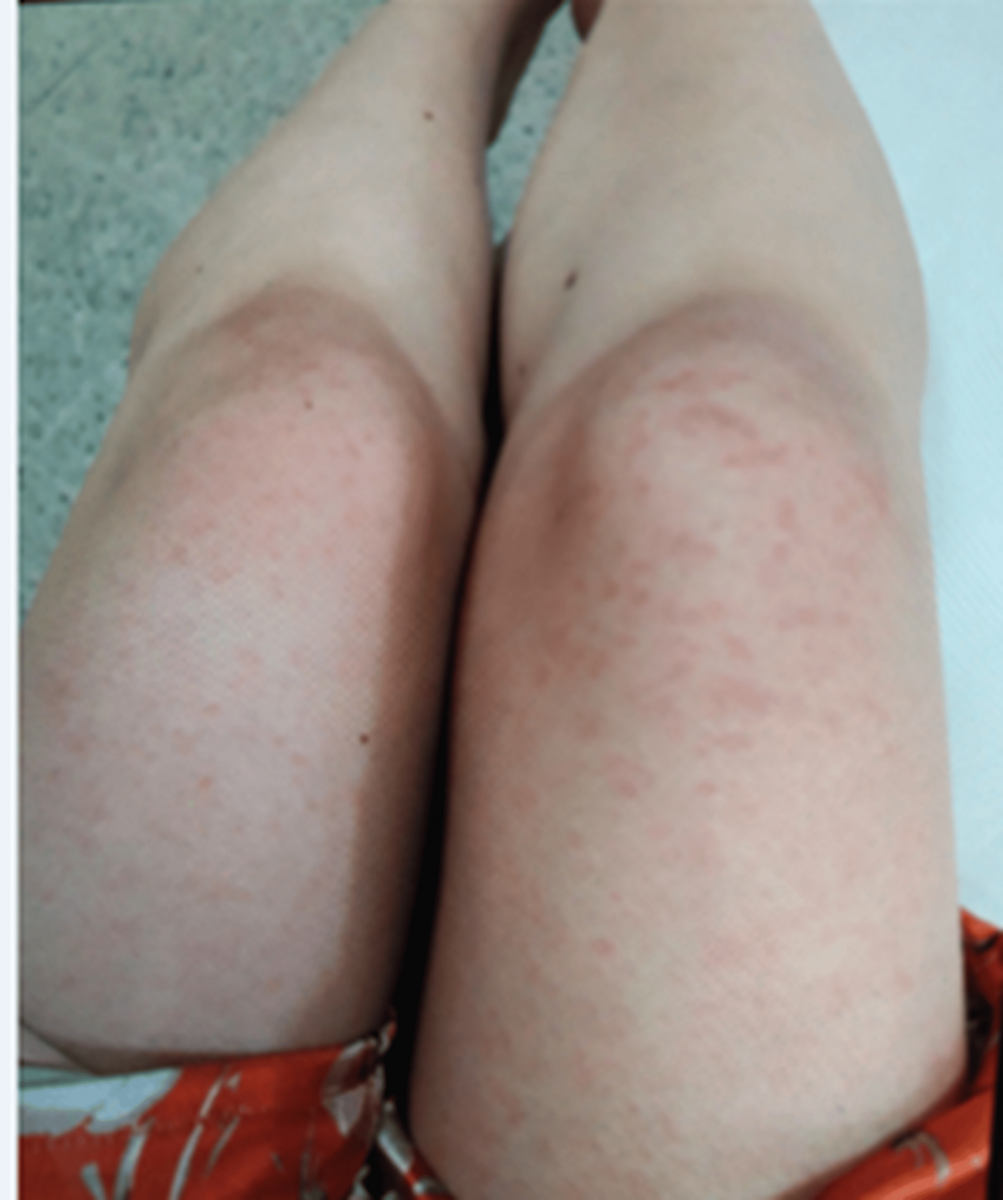
Erythema on her hands and knees that appeared along with the fever

She was readmitted two days later due to fever, general malaise, and once again erythema on her hands and knees that appeared along with the fever (see Figure 3).

**Figure 4:**
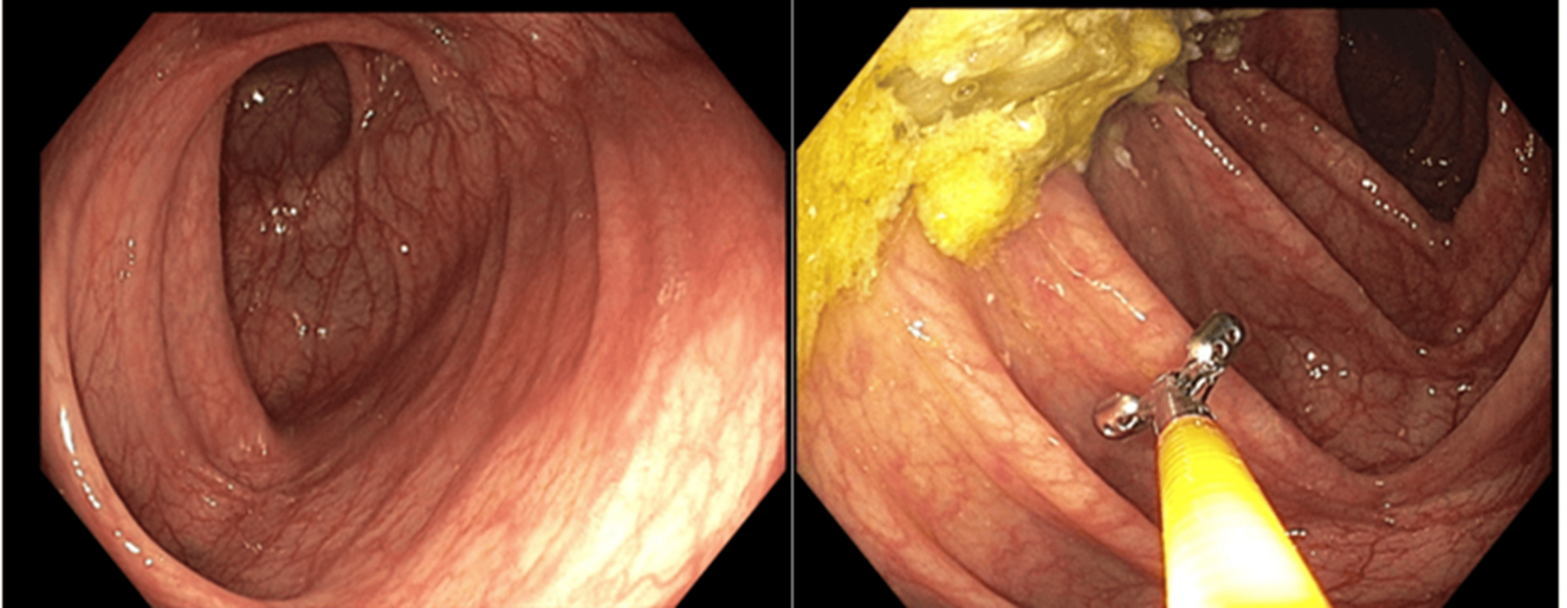
Contrasted abdominal tomography showed thickening of the mucosal layer of the transverse colon around the splenic flexure. Colonoscopy was performed, providing normal results

These symptoms appeared 12 hours after starting therapy with AZA. Physical examination registered a temperature of 38.3°C, a heart rate of 120 beats per minute, and erythematous macules on the thighs and hands. Paraclinical tests reported the following: normal leukocytes, renal function, liver profile, and comprehensive metabolic panel (CMP), platelet count of 349000, C-reactive protein of 3.7 mg/L, and procalcitonin of 0.71 ng/mL (see Table 1). AZA therapy was suspended, and tests ruled out infection by human immunodeficiency virus, cytomegalovirus, Epstein-Barr virus, human T-lymphotropic virus 1 (HTLV-1), *Salmonella* spp., *Treponema pallidum*, *Histoplasma capsulatum* spp., and *Strongyloides stercoralis*. A molecular stool panel detected enteroaggregative *Escherichia coli*, and a contrasted abdominal tomography showed thickening of the mucosal layer of the transverse colon around the splenic flexure. Hence, a colonoscopy was performed, providing normal results (see Figure 4).

AZA was then restarted at 100 mg/day, which led to a new temperature of 38°C and a heart rate of 115 beats per minute (see Figure 1: second hospitalization). When AZA was discontinued again, symptoms completely improved, so the patient was discharged after changing the immunosuppressive strategy to mycophenolate mofetil treatment.

This is a very particular and interesting case, as it displays a rare adverse effect associated with a medication commonly used by rheumatologists. If not identified in a timely manner, this condition can lead to multi-organ failure and potentially end the patient's life.

## Discussion

In clinical practice, AZA is used as a steroid-sparing medication for the treatment of several systemic autoimmune diseases such as inflammatory bowel disease (IBD), rheumatoid arthritis, vasculitis, pemphigus (bullous and vulgaris), myasthenia gravis, and SLE [5]. The main indication for the administration of AZA in patients with SLE is the kidney involvement, which is employed as maintenance therapy after induction therapy with cyclophosphamide [2]. Likewise, it is used as maintenance therapy in other forms of moderate or severe lupus, such as subacute cutaneous lupus, discoid lupus, cutaneous vasculitis, lupus pneumonitis, thrombocytopenia, and hemolytic anemia. AZA is also the preferred immunosuppressant for pregnant women but is contraindicated in breastfeeding [8].

AZA is a prodrug with no inherent immunosuppressive activity. It is an imidazole, derived from 6-mercaptopurine (6-MP), and is therefore classified as a purine analogue. After oral administration, it is almost completely absorbed in the intestine. It does not cross the blood-brain barrier but is well distributed throughout the body, having a mean lifetime of only about three hours due to non-enzymatic conversion to 6-MP. By action of the enzymes hypoxanthine-guanine phosphoribosyltransferase and thiopurine methyltransferase (TPMT), 6-MP is then converted to its active metabolites, mercaptopurine and thioguanine, which ultimately act by inhibiting RNA and DNA synthesis [1,9].

When TPMT activity decreases, dose-dependent adverse effects, such as myelosuppression or hepatotoxicity, may occur. These adverse effects can occur at any time during treatment, and their estimated incidence goes from 4% to 27% of treated patients. AZA can also produce non-dose-dependent adverse effects that are independent of TPMT activity, such as AHS. This idiosyncratic reaction, although less well known, is potentially life-threatening and requires early awareness, diagnosis, and prompt management [10].

AHS occurs in approximately 2% of patients within a few weeks of starting treatment, with most cases occurring within four weeks of onset [3]. While the age or sex of the patient does not appear to be associated with AHS incidence [10], it has been reported that those with IBD or multiple sclerosis may have an increased risk of developing it. The reasons are unclear but possibly explained by a genetic polymorphism that predisposes them to this manifestation [5]. AHS often mimics features commonly related to sepsis or resembles a relapse of the disease being treated, making early diagnosis difficult [7]. The most common systemic symptoms include fever, malaise, arthralgias, abdominal pain, nausea, vomiting, and, quite often, skin manifestations. According to a review by Bidinger et al., about 50% of 67 cases published between 1986 and 2009 displayed skin conditions, usually appearing a few days after systemic symptoms [5]. The most frequently reported cutaneous manifestations include maculopapular, vesicular, or pustular lesions, while purpura or erythema nodosum occur in a small percentage of patients [10]. The skin reaction predominantly reported in the literature is neutrophilic dermatosis [11]. Bidinger et al. conducted a case series where 76% (25/33) of patients who displayed cutaneous symptoms had biopsy or clinical features consistent with neutrophilic dermatosis, while the remaining 24% (8/33) were reported as unspecified cutaneous disease [5]. Less frequently, AHS can lead to liver [12] and renal dysfunction or even hypotension and shock, which was the case with our patient. It is noteworthy that laboratory findings report abnormalities such as neutrophilia, leukocytosis, anemia, elevated inflammatory markers, and, rarely, positive anti-neutrophil cytoplasmic antibodies [3]. While these features were not present in our case, the timing of the symptoms and the presence of rash alone were highly suggestive of AHS.

Regarding AHS pathophysiology, current studies have suggested an immune-mediated type III or IV reaction [3]. Whereas the 6-MP fraction of AZA and its metabolites are responsible for most of the dose-dependent side effects, the imidazole component could be responsible for the hypersensitivity reaction, as a hapten might be produced when imidazole binds to certain proteins. This theory is supported by several case reports of AZA hypersensitivity patients who were able to tolerate subsequent 6-MP administration. However, there are also case reports of hypersensitivity reactions to 6-MP both after initial administration and after re-exposure. Therefore, the exact mechanism of AHS remains unclear [5].

Recently, polymorphisms of NUDT15 [13,14] and their relationship to dose-dependent AZA adverse effects, especially leukopenia, have been identified in patients with IBD who were of Asian origin. Recent studies in other diseases and in other populations, including European populations, show that, also in these cases, such polymorphisms could be a risk marker for the accumulation of active metabolites [10]. Genetic polymorphisms in the inositol triphosphate pyrophosphatase gene may also be associated with hypersensitivity [5].

Further research is needed to confirm whether these polymorphisms could actually be useful in identifying patients at increased risk of developing AZA-related adverse effects [10]. This case demonstrates that infectious processes and an outbreak of the underlying disease must always be ruled out within the main differential diagnoses. In most published cases, an ongoing infectious process was the initial premise, and AHS was not considered for diagnosis until the skin condition appeared or a rapid clinical worsening occurred after the reintroduction of AZA [10]. Current evidence suggests that AHS should be considered as a differential diagnosis in any patient displaying general symptoms, fever, or skin lesions after the initiation of AZA [7,15]. Although Sweet's syndrome (SS) is one of the main differentials, Bidinger et al. found that 39% of patients (19/49) who were diagnosed with AHS were on corticosteroids at the time of the display of symptoms, which is atypical for SS, as corticosteroids are the treatment of choice. Moreover, the location of the lesions constitutes an additional element for the differential, as in SS they are more frequently located on the head, neck, and trunk and in AHS they appear with more frequency in the lower extremities [5].

Given the associated mortality, it is safest to discontinue the use of the medication. After discontinuation, the complete resolution of symptoms is usually expected to occur within a week, and the use of glucocorticoids is often unnecessary [3,16]. It is important to note that continuation or reintroduction of AZA can lead to multi-organ failure, including hepatic and renal dysfunction, severe hypotension, congestive heart failure, and cardiogenic shock, so once AHS has occurred, future re-exposure is contraindicated [10,16,17].

## Conclusions

AHS is a rare adverse effect not frequently reported in the literature. It is considered a life-threatening clinical phenomenon. Since its clinical presentation is often mistaken for an infectious process or a relapse of the disease being treated, early diagnosis is difficult; therefore, a high level of suspicion is required. Given the associated mortality, it is safest to discontinue the medication immediately, which is expected to result in the complete resolution of symptoms. Likewise, it should not be forgotten that future use is contraindicated because of the associated fatal outcomes.
